# PEELing: an integrated and user-centric platform for spatially resolved proteomics data analysis

**DOI:** 10.1093/bioinformatics/btaf439

**Published:** 2025-08-06

**Authors:** Xi Peng, Jody Clements, Zuzhi Jiang, Shuo Han, Stephan Preibisch, Jiefu Li

**Affiliations:** Janelia Research Campus, Howard Hughes Medical Institute, Ashburn, VA 20147, United States; Janelia Research Campus, Howard Hughes Medical Institute, Ashburn, VA 20147, United States; Janelia Research Campus, Howard Hughes Medical Institute, Ashburn, VA 20147, United States; Yuanpei College, Peking University, Beijing 100871, China; Key Laboratory of RNA Science and Engineering, Center for Excellence in Molecular Cell Science, Shanghai Institute of Biochemistry and Cell Biology, Chinese Academy of Sciences, Shanghai 200031, China; Janelia Research Campus, Howard Hughes Medical Institute, Ashburn, VA 20147, United States; Janelia Research Campus, Howard Hughes Medical Institute, Ashburn, VA 20147, United States

## Abstract

**Summary:**

Molecular compartmentalization is vital for cellular physiology. Spatially resolved proteomics allows biologists to survey protein composition and dynamics with subcellular resolution. Here, we present PEELing, an integrated package and user-friendly web service for analyzing spatially resolved proteomics data. PEELing assesses data quality using curated or user-defined references, performs cutoff analysis to remove contaminants, connects to databases for functional annotation, and generates data visualizations—providing a streamlined and reproducible workflow to explore spatially resolved proteomics data.

**Availability and implementation:**

PEELing and its tutorial are publicly available at https://peeling.janelia.org/ (Zenodo DOI: 10.5281/zenodo.15692517). A Python package of PEELing is available at https://github.com/JaneliaSciComp/peeling/ (Zenodo DOI: 10.5281/zenodo.15692434).

## 1 Introduction

**Figure 1.**
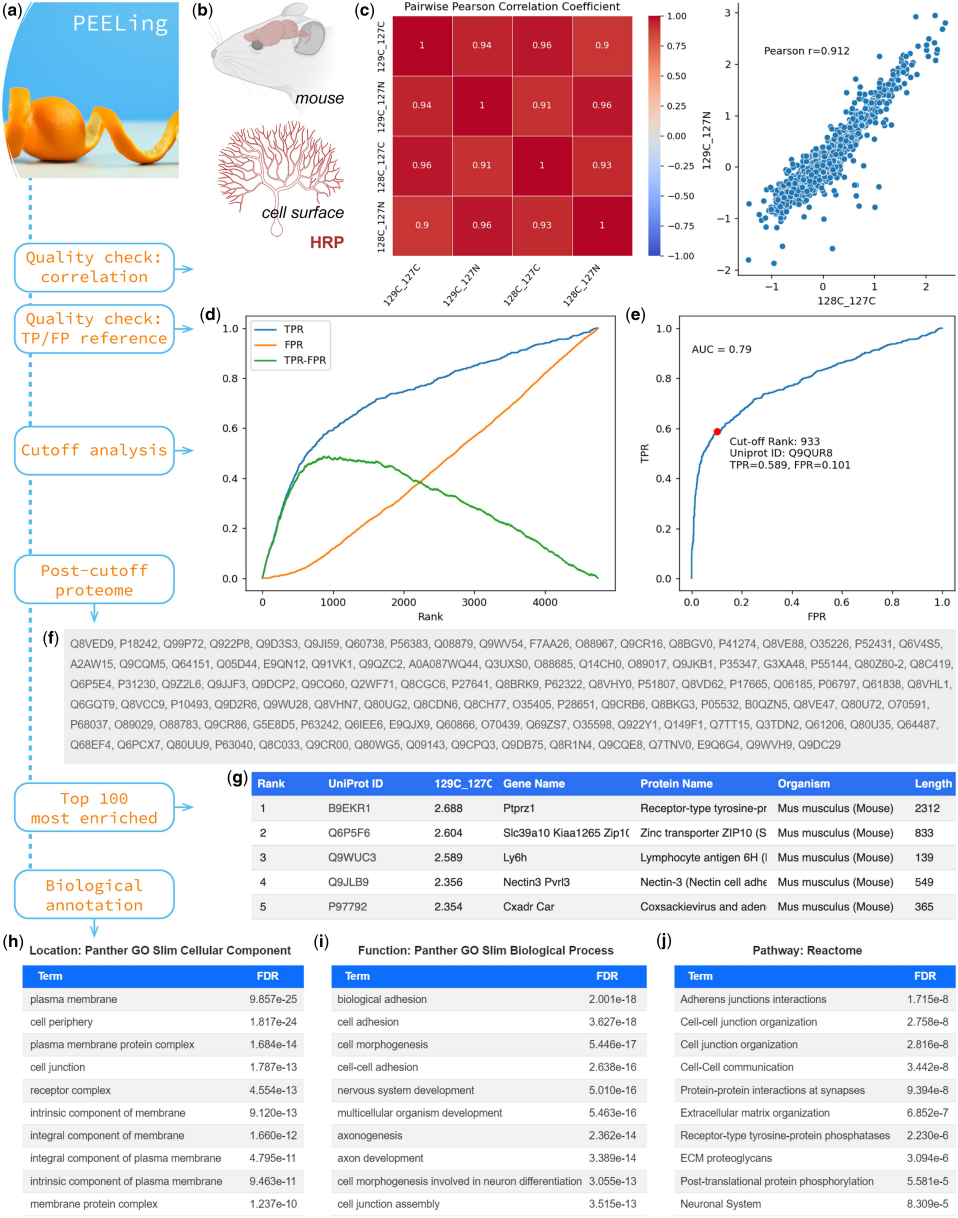
PEELing workflow and the analysis of a mouse cell-surface proteome. (a) PEELing workflow. (b) Shuster *et al.* (2022) used horseradish peroxidase (HRP)-mediated cell-surface biotinylation to capture the cell-surface proteome of mouse Purkinje cells at postnatal day 15. The authors used tandem mass tag (TMT) (Thompson *et al.* 2003) based quantitative mass spectrometry. 129C and 128C TMT tags were used for cell-surface-labeled samples, while 127C and 127N tags were used for non-labeled controls, yielding four labeled-to-control ratios (File 1, available as supplementary data at *Bioinformatics* online). (c) Correlation plots and coefficients. (d) True positive rate (TPR, blue), false positive rate (FPR, orange), and their difference (TPR–FPR, green) plotted against 129C:127N ratio-based ranking (*x*-axis). (e) Receiver operating characteristic (ROC) curve, based on ranking by 129C:127N. Red dot, cutoff position. (f) Post-cutoff proteome. (g) Top 5 most enriched proteins based on 129C:127C. On the website, this list extends to the top 100. Length unit: amino acids. (h–j) Protein ontology analyses for localization (h), function (i), and pathway (j). The PEELing icon was produced by DALL-E of OpenAI. AUC, area under the curve; FDR, false discovery rate.

Localization and function of proteins are always coupled. For instance, proteins for intercellular adhesion and communication are localized to the cell surface while many energy-producing enzymes stay in the mitochondrion. High-resolution, proteome-wide mapping of protein localization is of core importance for understanding cellular organization and processes. Emerging technologies for spatially resolved proteomics, particularly by proximity labeling, make this possible and broadly applicable in cell biology (Roux *et al.* 2012, Rhee *et al.* 2013, Loh *et al.* 2016, Branon *et al.* 2018, Han *et al.* 2018, Geri *et al.* 2020, Qin *et al.* 2021). Like other enrichment-based profiling methods, spatially resolved proteomics can be interfered by ineffective enrichment, nonspecific contamination, and other factors. Rigorous assessment of data quality and proper data processing is crucial for interpreting the results and for designing subsequent studies. However, this can be complex and overwhelming, particularly for biologists with limited proteomics experience or systems biology background. To address this, we built PEELing (*p*roteome *e*xtraction from *e*nzymatic *l*abel*ing* data), a platform integrating data quality checks, contaminant removal, functional annotation, and visualization into an automated workflow (Fig. 1a).

## 2 Implementation, benchmarks, and results

In Fig. 1, we used a published cell-surface proteome of mouse developing Purkinje cells (Shuster *et al.* 2022) (Fig. 1b and File 1, available as supplementary data at *Bioinformatics* online) to demonstrate the functionalities of PEELing. In this study (Shuster *et al.* 2022), cell-surface proteins were chemically labeled and isolated (“labelled” groups, hereafter) using horseradish peroxidase (HRP)-mediated cell-surface biotinylation (Loh *et al.* 2016, Li *et al.* 2020, Shuster *et al.* 2022). Non-labeled control groups (“control”) were included to capture contaminants such as endogenously biotinylated proteins and nonspecific binders to isolation reagents. PEELing uses a ratiometric strategy as previously described (Hung *et al.* 2014) in which the labeled-to-control ratio of each protein reflects whether this protein is cell-surface enriched or not. A *bona fide* cell-surface protein should exhibit a high ratio because it should be enriched in the labeled group relative to the control group. A contaminant should have a low ratio since it should be captured similarly in both the labeled and control groups.

To assess data quality, PEELing started with pairwise correlation analysis to check whether biological replicates exhibited consistency (Fig. 1c). To determine whether cell-surface proteins were enriched, PEELing ranked all detected proteins in descending order based on the labeled-to-control ratio and scanned through them to mark curated cell-surface proteins (true positives, TPs; File 10, available as supplementary data at *Bioinformatics* online) and intracellular contaminants (false positives, FPs; File 12, available as supplementary data at *Bioinformatics* online). As shown in Fig. 1d, true positive rate (TPR, blue), false positive rate (FPR, orange), and their difference (TPR–FPR, green) were calculated and plotted against ratio-based ranking. TPR increased quickly, while FPR rose slowly, which led to a single peak of TPR–FPR, revealing a high ranking and an effective enrichment of cell-surface proteins. This is further illustrated by a receiver operating characteristic (ROC) curve bending toward the left-upper corner and exhibiting an area under the curve (AUC) of 0.79 (Fig. 1e). An AUC greater than 0.7 generally indicates acceptable enrichment, whereas an AUC near 0.5 suggests random capture—implying that either the proximity labeling reaction or the protein enrichment procedure has failed and requires further optimization.

To test whether PEELing detects data of poor quality, we generated a pseudo dataset with random values mimicking a failed enrichment (File 2, available as supplementary data at *Bioinformatics* online and Fig. 1, available as supplementary data at *Bioinformatics* online). TPR, FPR, and ROC curves all followed the diagonal line (Fig. 1b, c, e, and f, available as supplementary data at *Bioinformatics* online), showing a complete mix of cell-surface and intracellular proteins without any enrichment (Fig. 1d and g, available as supplementary data at *Bioinformatics* online). We then tested reference specificity by analyzing the cell-surface proteome (Shuster *et al.* 2022) (File 1, available as supplementary data at *Bioinformatics* online) with nuclear and mitochondrial references (Files 14–17, available as supplementary data at *Bioinformatics* online). No enrichment of proteins of unintended cellular compartments (Fig. 2, available as supplementary data at *Bioinformatics* online) showed the quality of the example data as well as the necessity of data-reference matching in PEELing analysis.

All analyses above used the labeled-to-control ratio as the enrichment indicator. Protein abundance is widely used in proteomic data quantification; however, it is not a reliable enrichment indicator in spatially resolved profiling compared with the labeled-to-control ratio (Fig. 3, available as supplementary data at *Bioinformatics* online, compared with Fig. 1). From the same cell-surface proteome dataset (Shuster *et al.* 2022), we obtained protein abundance values of the labeled groups (File 3, available as supplementary data at *Bioinformatics* online). Despite comparable correlations based on abundance (Fig. 3a, available as supplementary data at *Bioinformatics* online) and labeled-to-control ratio (Fig. 1c), cell-surface protein enrichment was not observed when ranking the proteome by abundance (Fig. 3b, c, e, and f, available as supplementary data at *Bioinformatics* online). Intracellular proteins were highly enriched instead (Fig. 3d and g, available as supplementary data at *Bioinformatics* online). Notably, a contaminant (e.g. a nonspecific binder to the isolation reagent) can be abundant while a cell-surface protein may have a low expression level. As long as the control group captures the contaminant but not the cell-surface protein, these proteins will be ranked correctly by the labeled-to-control ratio instead of being ranked reversely by abundance. Therefore, labeled-to-control ratio is the preferred input data for PEELing.

Following data quality checks, PEELing performed cutoff analysis to remove contaminants. For each labeled-to-control ratio, PEELing found the ranking position where TPR–FPR was maximal, as indicated by the peak of the green line in Fig. 1d and the red dot in Fig. 1e, and retained proteins ranked above this position. The “TPR–FPR maximum” cutoff provides two key benefits: (i) the cutoff position is determined by data quality rather than an arbitrary value, allowing for unbiased assessment of the proteomic results. If the data are of high quality with sparse FPs, TPR–FPR will peak later in the ranking, resulting in the retention of more proteins. If the data are heavily contaminated with FPs, TPR–FPR will peak earlier, leading to the retention of fewer proteins. (ii) Any protein ranked above the cutoff position is retained, regardless of how it is annotated by a database. Therefore, missing annotations or occasional incorrect annotations in databases will not impact the analysis, as long as they are largely accurate and comprehensive. As illustrated in Fig. 4, available as supplementary data at *Bioinformatics* online, 10-fold reduction of reference coverage, in either TP/FP or both, did not impair the analysis of the cell-surface proteome of Purkinje cells (compared with Fig. 1). Additionally, the *Drosophila* proteome has less complete annotations than those of mouse and human, leading to jagged TPR, FPR, and ROC curves (Fig. 5c and d, available as supplementary data at *Bioinformatics* online). Nevertheless, it did not interfere with the analysis of a *Drosophila* neuronal surface proteome (Li *et al.* 2020) (Fig. 5, available as supplementary data at *Bioinformatics* online and File 4, available as supplementary data at *Bioinformatics* online). Importantly, in certain physiological or pathological contexts, intracellular proteins can be transported to the cell surface—for instance, RNA helicase U5 snRNP200 in acute myeloid leukemia (Knorr *et al.* 2023). PEELing retains these proteins if they are ranked highly, potentially enabling researchers to discover novel biomarkers and cellular processes. Despite its robustness to varied reference coverages, we note that PEELing relies on high-quality references (see Supplementary Materials—Method Details, available as supplementary data at *Bioinformatics* online on how to create references). When references are not possible to obtain, commonly used statistical analysis is an alternative approach.

PEELing conducted cutoff analysis on all submitted labeled-to-control ratios individually and, for the final proteome, retained only those proteins that passed the cutoff of all ratios, which further removed contaminants. As shown in Fig. 1f, PEELing displayed the post-cutoff proteome and provided information of the top 100 most enriched proteins for each labeled-to-control ratio (Fig. 1g). On the website, each UniProt accession number is a clickable link to the corresponding UniProt protein page (UniProt Consortium 2023). PEELing then transmitted the post-cutoff proteome to the Panther server (Mi *et al.* 2019, Thomas *et al.* 2022) for over-representation analyses on protein localization (Fig. 1h), function (Fig. 1i), and pathway (Fig. 1j) and revealed an enrichment of cell-surface proteins related to cell adhesion and neuronal development, perfectly matching the dataset—a cell-surface proteome of developing Purkinje cells.

Simply plugging in corresponding references, PEELing is ready for analyzing spatially resolved proteomics data of any cellular compartments, such as the nucleus (Fig. 6, available as supplementary data at *Bioinformatics* online) by TurboID (Branon *et al.* 2018) (File 5, available as supplementary data at *Bioinformatics* online) and miniTurbo (Branon *et al.* 2018) (File 6, available as supplementary data at *Bioinformatics* online), as well as the mitochondrion (Figs 7 and 8, available as supplementary data at *Bioinformatics* online) by APEX2 (Lam *et al.* 2015, Han *et al.* 2017) (File 7, available as supplementary data at *Bioinformatics* online) and BioID (Roux *et al.* 2012, Branon *et al.* 2018, Antonicka *et al.* 2020) (Files 8 and 9, available as supplementary data at *Bioinformatics* online). Together, we demonstrated that PEELing is widely applicable to: (i) various organisms, such as human (Figs 6–8, available as supplementary data at *Bioinformatics* online), mouse (Fig. 1), and fruit fly (Fig. 5, available as supplementary data at *Bioinformatics* online); (ii) diverse subcellular compartments, including membrane-enclosed organelles (nucleus and mitochondrion) and open space (cell surface); (iii) all commonly used proximity labeling tools, including peroxidases (APEX2 and HRP) and biotin ligases (BioID, TurboID, and miniTurbo); and (iv) different mass spectrometry quantification methods including label free (Fig. 8, available as supplementary data at *Bioinformatics* online) and isobaric labeling (Fig. 1 and Figs 5–7, available as supplementary data at *Bioinformatics* online). Moreover, PEELing features a plug-n’-play web service for a complete workflow (Fig. 1a) including data visualization—all data panels shown here were directly from PEELing—as well as a Python package enabling advanced customization and integration with other bioinformatics tools. It thus provides a user-friendly yet versatile platform for exploring subcellular organization of the proteome.

## Supplementary Material

btaf439_Supplementary_Data

## Data Availability

All datasets used for benchmarking are included as Files 1–17, available as supplementary data at *Bioinformatics* online.
