# Supplementary material for: PEELing: an integrated and user-centric platform for spatially resolved proteomics data analysis
Source: Bioinformatics. 2025 Aug 6;41(8):btaf439. doi: 10.1093/bioinformatics/btaf439 (PMC12343102; doi:10.1093/bioinformatics/btaf439)
Supplement: btaf439_Supplementary_Data [file btaf439_supplementary_data.zip › PEELing_SupplementaryMaterials.pdf]

Supplementary Materials

Method Details

Data input

We note that PEELing does not handle raw mass spectral data or provide custom searches (e.g., labeling site identification or non-canonical translation analysis), which needs to be processed by peptide identification and quantification software such as MaxQuant (Cox and Mann, 2008), FragPipe (Kong *et al.*, 2017), Spectrum Mill (Broad Institute), or Proteome Discoverer (Thermo Fisher). The PEELing website and command line program accept tab-separated value (.tsv) files, which contain UniProt accession numbers in their first columns and enrichment indexes in the remaining columns (e.g., **Supplementary File 1–9**). PEELing uses UniProt accession numbers as protein identifiers and updates user input to the current UniProt version to help database searches. As benchmarked and discussed above, labelled-to-control ratio is the preferred enrichment index; however, users may choose other indexes according to their experimental design.

True positive and false positive references

We constructed true positive (TP) and false positive (FP) references using SwissProt-reviewed annotations. The PEELing web service automatically updates these references from the UniProt database (Consortium *et al.*, 2023), ensuring that they remain current and accurate.

Cell-surface TP reference (**Supplementary File 10**) is specified by the UniProt term: *((cc_scl_term:SL-0112) OR (cc_scl_term:SL-0243) OR (keyword:KW-0732) OR (cc_scl_term:SL-9906) OR (cc_scl_term:SL-9907)) AND (reviewed:true)*, which includes SwissProt-reviewed extracellular (SL-0112), secreted (SL-0243), signal peptide-containing (KW-0732), type II transmembrane (SL-9906), or type III transmembrane (SL-9907) proteins. Cell-surface FP reference (**Supplementary File 12**) is specified by the UniProt term: *(((cc_scl_term:SL-0091) OR (cc_scl_term:SL-0173) OR (cc_scl_term:SL-0191)) AND (reviewed:true)) NOT (((cc_scl_term:SL-0112) OR (cc_scl_term:SL-0243) OR (keyword:KW-0732) OR (cc_scl_term:SL-9906) OR (cc_scl_term:SL-9907)) AND (reviewed:true))*, including SwissProt-reviewed cytosolic (SL-0091), mitochondrial (SL-0173), and nuclear (SL-0191) proteins that do not express on the cell surface. Some cell-surface proteins, such as the Notch family proteins, are also localized in intracellular compartments and are not considered false positives, and are thus removed from the FP reference.

Nuclear TP reference (**Supplementary File 14**) is specified by the UniProt term: *(cc_scl_term:SL-0191) AND (reviewed:true)*, which includes SwissProt-reviewed nuclear proteins

(SL-0191). Nuclear FP reference (**Supplementary File 15**) is specified by the UniProt term: *(((cc_scl_term:SL-0091) OR (cc_scl_term:SL-0173) OR (cc_scl_term:SL-0112) OR (cc_scl_term:SL-0243) OR (keyword:KW-0732) OR (cc_scl_term:SL-9906) OR (cc_scl_term:SL-9907)) AND (reviewed:true)) NOT ((cc_scl_term:SL-0191) AND (reviewed:true))*, including SwissProt-reviewed cytosolic (SL-0091), mitochondrial (SL-0173), and cell-surface (cell-surface TP term) proteins that do not express in the nucleus.

Mitochondrial TP reference (**Supplementary File 16**) is specified by the UniProt term: *(cc_scl_term:SL-0173) AND (reviewed:true)*, which includes SwissProt-reviewed mitochondrial proteins (SL-0173). Mitochondrial FP reference (**Supplementary File 17**) is specified by the UniProt term: *(((cc_scl_term:SL-0091) OR (cc_scl_term:SL-0191) OR (cc_scl_term:SL-0112) OR (cc_scl_term:SL-0243) OR (keyword:KW-0732) OR (cc_scl_term:SL-9906) OR (cc_scl_term:SL-9907)) AND (reviewed:true)) NOT ((cc_scl_term:SL-0173) AND (reviewed:true))*, including SwissProt-reviewed cytosolic (SL-0091), nuclear (SL-0191), and cell-surface (cell-surface TP term) proteins that do not express in the mitochondrion.

For custom TP and FP references, PEELing requests two tab-separated value (.tsv) files from the user, each containing one column of UniProt accession numbers (e.g., **Supplementary File 10–17**). Despite the robustness of this algorithm and its tolerance to imperfect references (**Supplementary Figure 4**), constructing high-quality references is essential for proper data filtering and interpretation. The TP reference should contain proteins known to be in the designated cellular compartment while the FP reference should contain proteins that are, to one's best knowledge, not in this compartment. Curated Swiss-Prot/UniProt and Gene Ontology Cellular Component (GOCC) databases, as well as relevant literature, provide resources for creating the TP and FP references. More TP and FP examples and their designing rules can be found in (Hung *et al.*, 2016) and (Cho *et al.*, 2020).

Reference-based data quality check and cutoff analysis

For each enrichment index (e.g., each labelled-to-control ratio), PEELing first ranks all proteins in descending order according to this index. For each protein on the ranked list, accumulated true positive count and false positive count above this ranking position are calculated to obtain true positive rate (TPR), false positive rate (FPR), and their difference (TPR–FPR) at this ranking position (visualized in **Figure 1d** and other plots of TPR, FPR, and TPR–FPR). A receiver operating characteristic (ROC) curve is produced accordingly (**Figure 1e** and other ROC curves).

For each enrichment index, the cutoff is set where TPR–FPR maximizes, representing the largest segregation of intended signal (TP) and unintended noise (FP). PEELing conducts cutoff

analysis on all submitted enrichment indexes individually and, for the final proteome, retains only those proteins passing the cutoff of all or multiple indexes. Both the PEELing website and command line program offer the optional "Tolerance" setting, enabling users to control the stringency of the cutoff. By default, it is set to 0, meaning that a protein must pass the cutoff of all indexes to be included in the final proteome. If it is set to n, a protein can fail the cutoff in up to n indexes and still be included in the final proteome. Despite the flexibility, we recommend setting the tolerance value to a small number to better filter out contaminants.

Protein annotation

From the post-cutoff proteome, PEELing retrieves from UniProt and displays gene names, protein names, organisms, and protein lengths of the top 100 enriched proteins based on each index. For protein ontology, PEELing sends the post-cutoff proteome to the Panther server (Thomas *et al.*, 2022; Mi *et al.*, 2019) for over-representation analysis. The annotation datasets used include: GO slim cellular component, for protein localization; GO slim biological process, for protein function; and reactome pathway, for signaling pathway. Results are ranked in ascending order by false discovery rate (FDR). Top 10 terms are listed along with their FDRs.

Supplementary Figures

Random values: simulating failed enrichment of cell-surface proteins

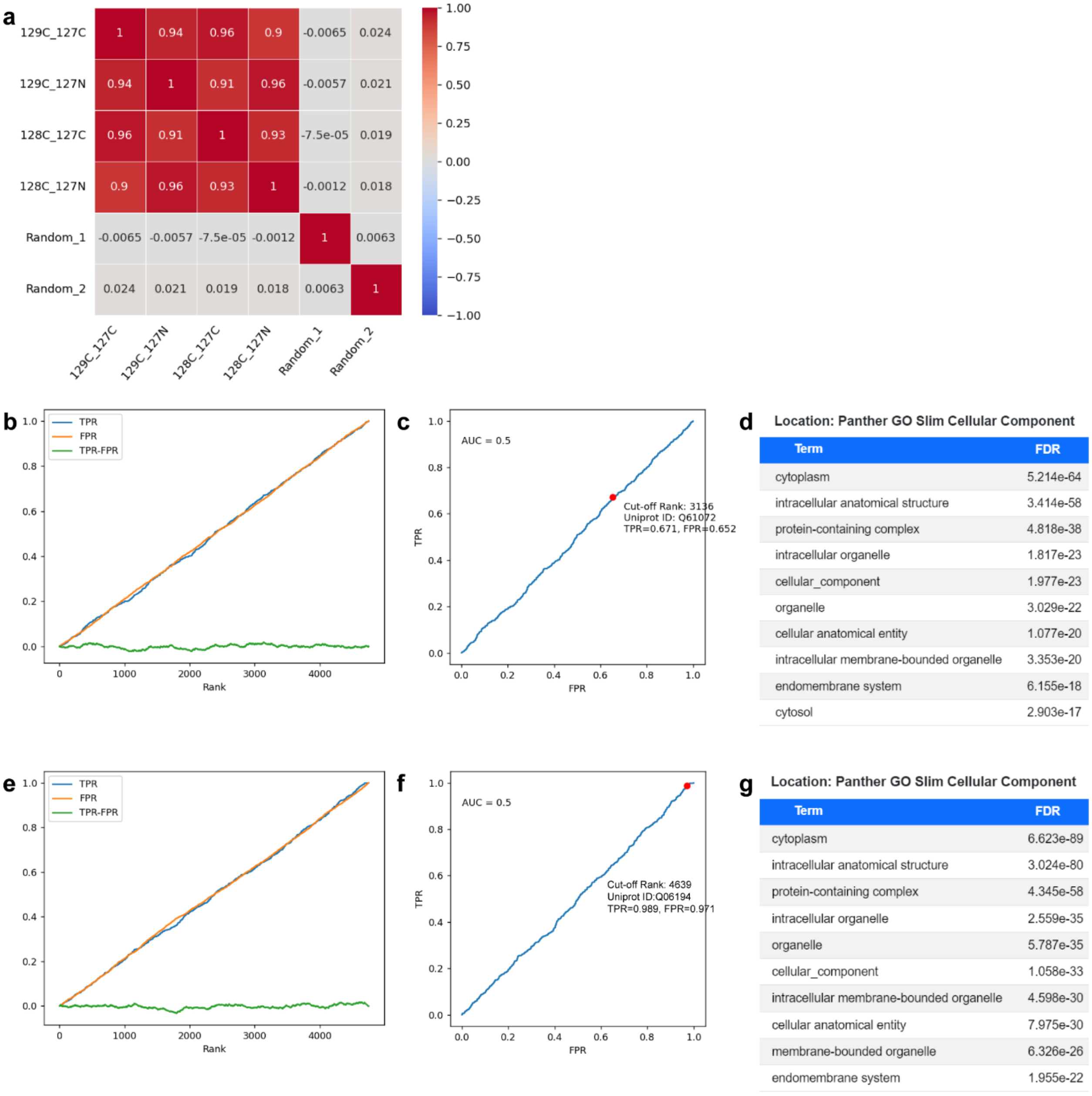

Supplementary Figure 1. Testing PEELing with randomized data.

To simulate failed enrichment, two sets of random values Random_1 and Random_2 were generated and added to the data of Purkinje cell cell-surface proteome (**Supplementary File 2**). (**a**) Correlation coefficients showing no correlation between the real and randomized data. (**b**,**e**) True positive rate (TPR, blue), false positive rate (FPR, orange), and their difference (TPR–FPR,

green) plotted against Random_1 (**b**) and Random_2 (**e**) based ranking (x-axis). (**c**,**f**) Receiver operating characteristic (ROC) curves, based on Random_1 (**c**) and Random_2 (**f**), respectively. AUC, area under the curve. (**d**,**g**) Subcellular localization annotation did not enrich any cell surface related terms. **d**, Random_1; **g**, Random_2. FDR, false discovery rate.

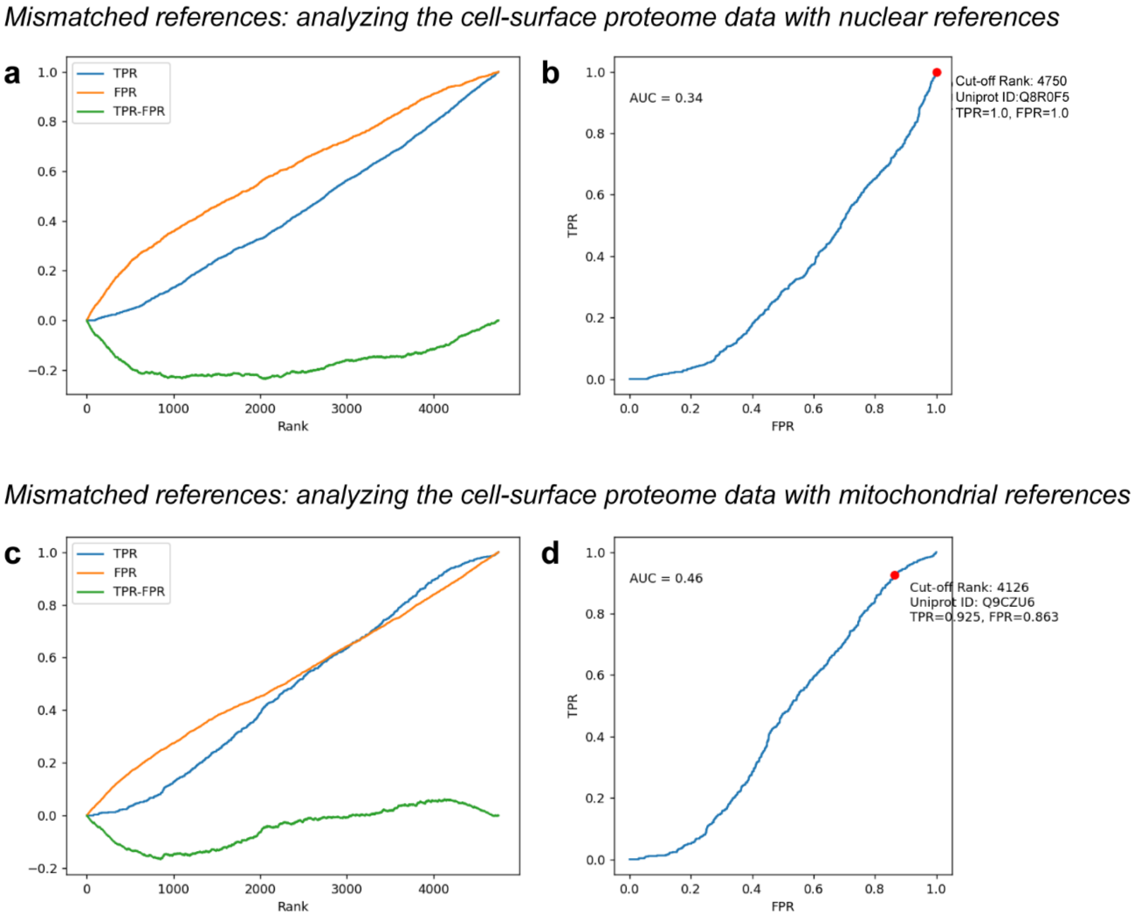

Supplementary Figure 2. Testing PEELing with mismatched references.

The cell-surface proteome of Purkinje cells (**Supplementary File 1**) was analyzed using mismatched references: nucleus (**a**,**b**; **Supplementary File 14**,**15**) and mitochondrion (**c**,**d**; **Supplementary File 16,17**). (**a**,**c**) True positive rate (TPR, blue), false positive rate (FPR, orange), and their difference (TPR–FPR, green) plotted against 129C:127N ratio-based ranking (x-axis). (**b**,**d**) Receiver operating characteristic (ROC) curves, based on ranking by 129C:127N. AUC, area under the curve.

Analysis based on protein abundances of the labelled groups, instead of labelled-to-control ratios

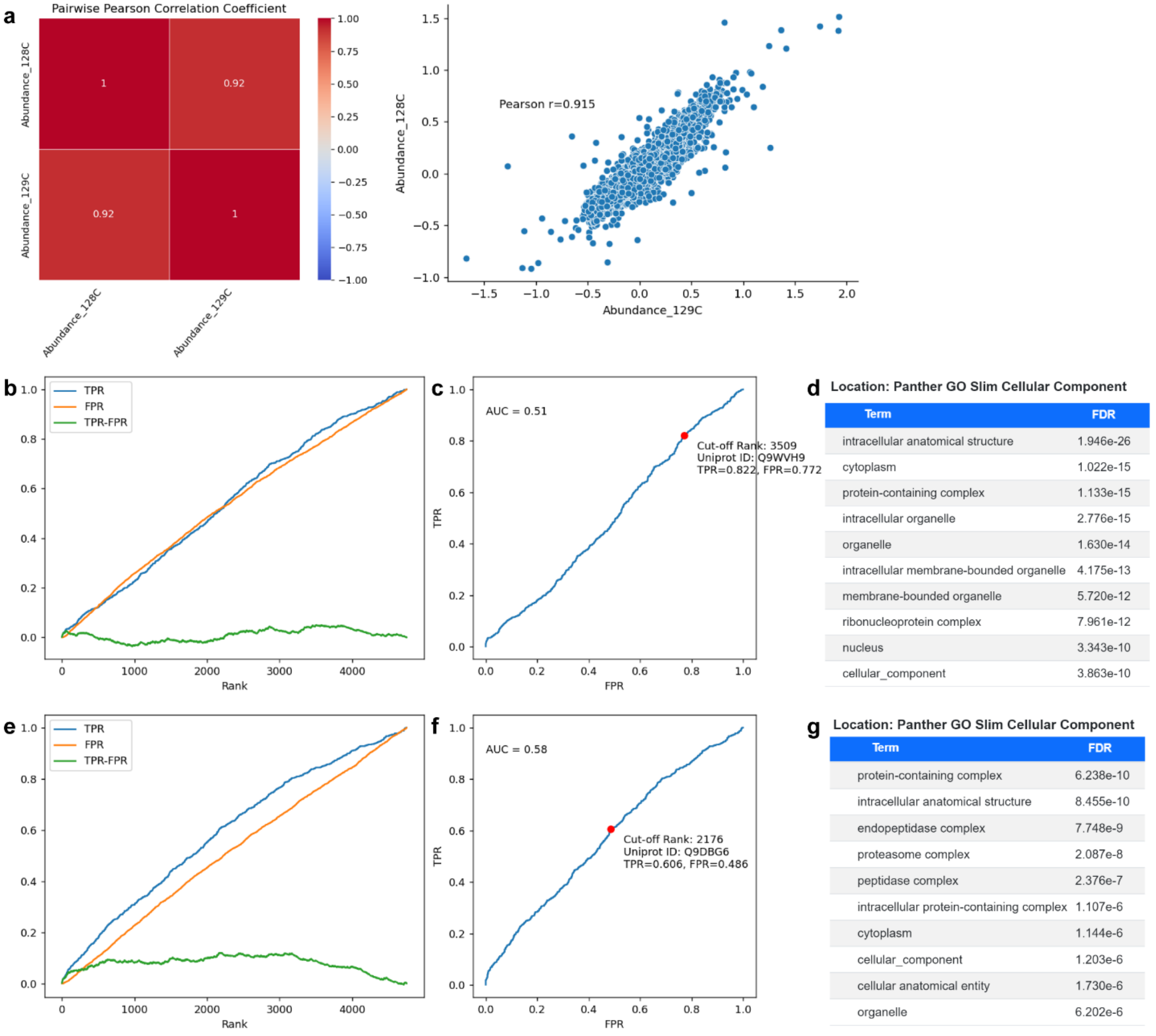

Supplementary Figure 3. Abundance is not a reliable indicator of enrichment.

Protein abundance data of cell-surface-labelled samples (TMT tags 128C and 129C) was obtained from the cell-surface proteome of Purkinje cells (Shuster *et al.*, 2022) (**Supplementary File 3**). (**a**) Correlation plots and coefficients. (**b**,**e**) True positive rate (TPR, blue), false positive rate (FPR, orange), and their difference (TPR–FPR, green) plotted against abundance-based ranking (x-axis). **b**, 128C; **e**, 129C. (**c**,**f**) Receiver operating characteristic (ROC) curves, based on ranking by abundance. **c**, 128C; **f**, 129C. AUC, area under the curve. (**d**,**g**) Subcellular localization of the abundance-ranked top 500 proteins. **d**, 128C; **g**, 129C. FDR, false discovery rate.

a

TP reference: full

FP reference: 10%

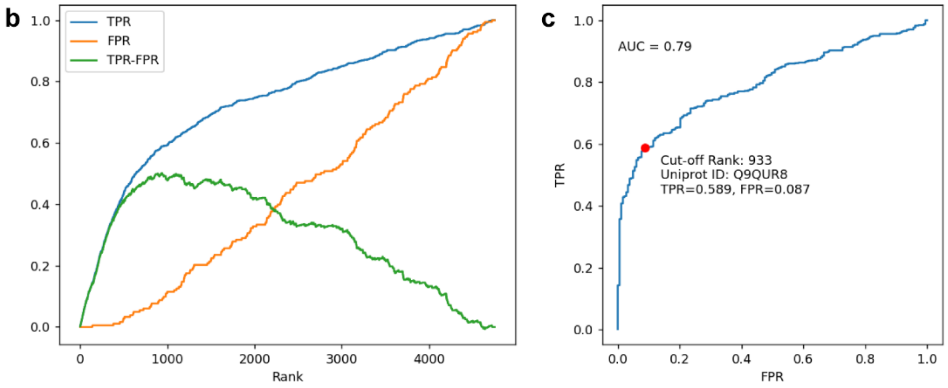

d Location: Panther GO Slim Cellular Component

Term	FDR
plasma membrane protein complex	5.095e-15
cell junction	5.383e-15
receptor complex	4.129e-13
membrane	7.830e-11
membrane protein complex	1.746e-10
endomembrane system	6.249e-10
cell periphery	2.823e-9
endoplasmic reticulum	3.042e-9
adherens junction	3.729e-9
plasma membrane	3.912e-9

e Function: Panther GO Slim Biological Process

Term	FDR
cell-cell adhesion	1.920e-24
cell adhesion	2.366e-24
cell morphogenesis	4.625e-23
multicellular organism development	1.509e-22
nervous system development	8.775e-20
axonogenesis	1.322e-17
cell morphogenesis involved in neuron differentiation	3.008e-17
cell morphogenesis involved in differentiation	5.597e-17
neurogenesis	6.325e-17
system development	7.247e-17

f Pathway: Reactome

Term	FDR
Cell junction organization	1.188e-7
Adherens junctions interactions	1.422e-7
Cell-Cell communication	1.633e-7
Protein-protein interactions at synapses	2.707e-7
Cell-cell junction organization	2.756e-7
Extracellular matrix organization	5.671e-7
Receptor-type tyrosine-protein phosphatases	2.766e-6
ECM proteoglycans	2.388e-5
Post-translational protein phosphorylation	6.873e-5
Neuronal System	8.284e-5

g

TP reference: 10%

FP reference: full

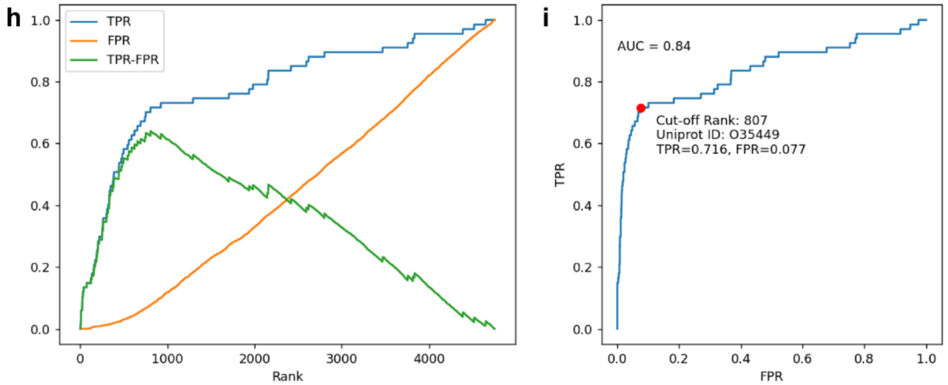

j Location: Panther GO Slim Cellular Component

Term	FDR
plasma membrane protein complex	6.527e-17
cell junction	1.388e-14
receptor complex	5.483e-14
cell periphery	6.311e-11
membrane	6.701e-11
membrane protein complex	1.232e-10
plasma membrane	2.100e-10
plasma membrane signaling receptor complex	9.665e-10
endoplasmic reticulum	1.001e-8
adherens junction	1.045e-8

k Function: Panther GO Slim Biological Process

Term	FDR
multicellular organism development	3.405e-25
cell adhesion	3.715e-25
cell morphogenesis	4.147e-25
cell-cell adhesion	5.105e-25
nervous system development	7.785e-23
axonogenesis	1.243e-19
neurogenesis	1.290e-19
system development	1.466e-19
cell morphogenesis involved in neuron differentiation	3.051e-19
cell morphogenesis involved in differentiation	6.023e-19

l Pathway: Reactome

Term	FDR
Adherens junctions interactions	1.776e-7
Cell junction organization	3.533e-7
Cell-cell junction organization	3.788e-7
Cell-Cell communication	3.840e-7
Protein-protein interactions at synapses	8.710e-7
Extracellular matrix organization	2.892e-6
ECM proteoglycans	1.496e-5
Receptor-type tyrosine-protein phosphatases	2.171e-5
Neuronal System	2.660e-4
Post-translational protein phosphorylation	7.147e-4

m

TP reference: 10%

FP reference: 10%

n

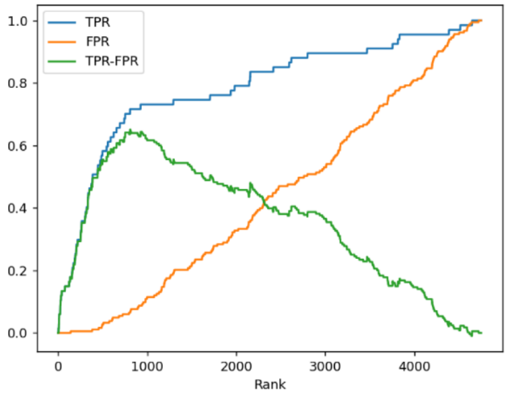

o

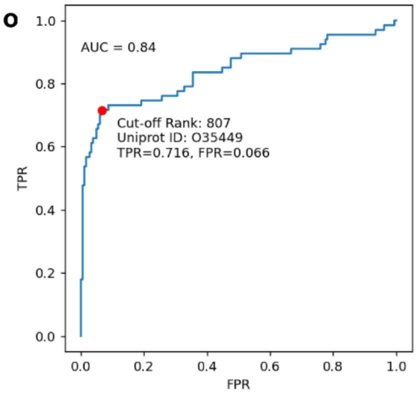

p Location: Panther GO Slim Cellular Component

Term	FDR
plasma membrane protein complex	6.527e-17
cell junction	1.388e-14
receptor complex	5.483e-14
cell periphery	6.311e-11
membrane	6.701e-11
membrane protein complex	1.232e-10
plasma membrane	2.100e-10
plasma membrane signaling receptor complex	9.665e-10
endoplasmic reticulum	1.001e-8
adherens junction	1.045e-8

q Function: Panther GO Slim Biological Process

Term	FDR
multicellular organism development	3.405e-25
cell adhesion	3.715e-25
cell morphogenesis	4.147e-25
cell-cell adhesion	5.105e-25
nervous system development	7.785e-23
axonogenesis	1.243e-19
neurogenesis	1.290e-19
system development	1.466e-19
cell morphogenesis involved in neuron differentiation	3.051e-19
cell morphogenesis involved in differentiation	6.023e-19

r Pathway: Reactome

Term	FDR
Adherens junctions interactions	1.776e-7
Cell junction organization	3.533e-7
Cell-cell junction organization	3.788e-7
Cell-Cell communication	3.840e-7
Protein-protein interactions at synapses	8.710e-7
Extracellular matrix organization	2.892e-6
ECM proteoglycans	1.496e-5
Receptor-type tyrosine-protein phosphatases	2.171e-5
Neuronal System	2.660e-4
Post-translational protein phosphorylation	7.147e-4

Supplementary Figure 4. Testing PEELing with coverage-reduced references.

We randomly removed 90% of genes from the cell-surface true-positive (TP) and false-positive (FP) references (**Supplementary File 10,12**) to produce truncated references with only 10% of the original coverage (**Supplementary File 11,13**) and then analyzed the cell-surface proteome of Purkinje cells (**Supplementary File 1**) using the truncated references (**m**–**r**) or pairing a full reference with a truncated one (**a**–**l**). (**b**,**h**,**n**) True positive rate (TPR, blue), false positive rate (FPR, orange), and their difference (TPR–FPR, green) plotted against 129C:127N ratio-based ranking (x-axis). (**c**,**i**,**o**) Receiver operating characteristic (ROC) curve, based on ranking by 129C:127N. Red dot, cutoff position. AUC, area under the curve. (**d**–**f**, **j**–**l**, **p**–**r**) Protein ontology analyses of post-cutoff proteomes for localization (**d**,**j**,**p**), function (**e**,**k**,**q**), and pathway (**f**,**l**,**r**). FDR, false discovery rate.

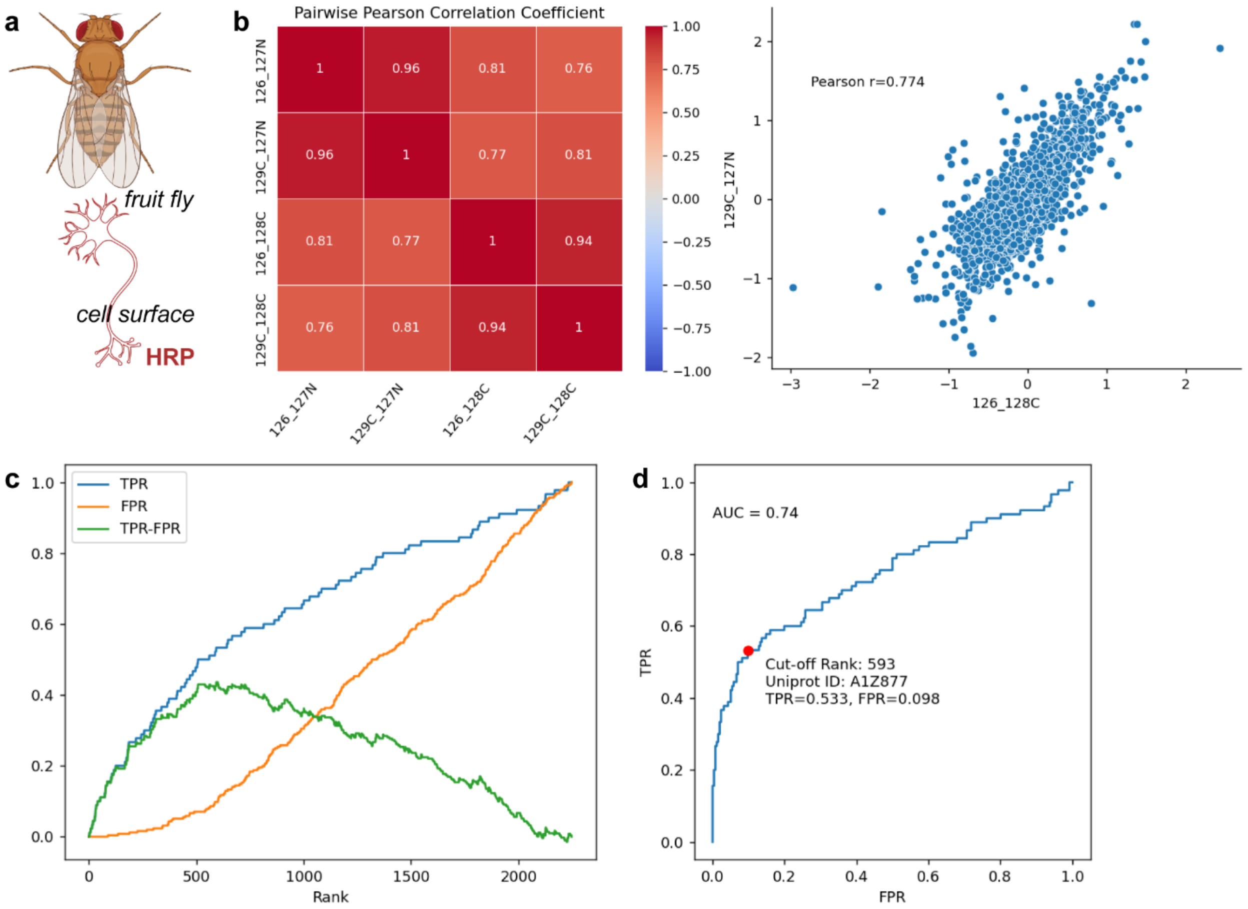

e Location: Panther GO Slim Cellular Component

Term	FDR
plasma membrane	5.591e-26
cell periphery	1.132e-24
integral component of membrane	8.571e-14
intrinsic component of membrane	1.134e-13
integral component of plasma membrane	1.499e-13
intrinsic component of plasma membrane	1.622e-13
membrane	4.537e-13
neuron projection	1.631e-11
cellular anatomical entity	5.007e-11
cellular_component	1.164e-10

f Function: Panther GO Slim Biological Process

Term	FDR
localization	5.470e-10
transport	1.107e-9
establishment of localization	1.195e-9
ion transport	4.469e-8
cell-cell signaling	1.933e-7
signaling	1.032e-6
cell communication	1.489e-6
anterograde trans-synaptic signaling	2.133e-6
trans-synaptic signaling	2.327e-6
chemical synaptic transmission	2.559e-6

g Pathway: Reactome

Term	FDR
Neuronal System	5.126e-10
Transmission across Chemical Synapses	2.383e-9
Transport of small molecules	9.498e-8
SLC-mediated transmembrane transport	1.430e-7
Axon guidance	1.351e-4
Nervous system development	1.394e-4
Neurotransmitter release cycle	1.560e-4
Metabolism	3.356e-4
Signal Transduction	4.724e-4
Developmental Biology	5.022e-4

Supplementary Figure 5. PEELing analysis of a *Drosophila* cell-surface proteome.

(**a**) The cell-surface proteome of *Drosophila* mature olfactory projection neurons was profiled by horseradish peroxidase (HRP) based cell-surface proteomics (Li *et al.*, 2020) (**Supplementary File 4**). (**b**) Correlation plots and coefficients. (**c**) True positive rate (TPR, blue), false positive rate (FPR, orange), and their difference (TPR–FPR, green) plotted against 129C:127N ratio-based ranking (x-axis). (**d**) Receiver operating characteristic (ROC) curve, based on ranking by

129C:127N. Red dot, cutoff position. AUC, area under the curve. (**e–g**) Protein ontology analyses for localization (**e**), function (**f**), and pathway (**g**). FDR, false discovery rate.

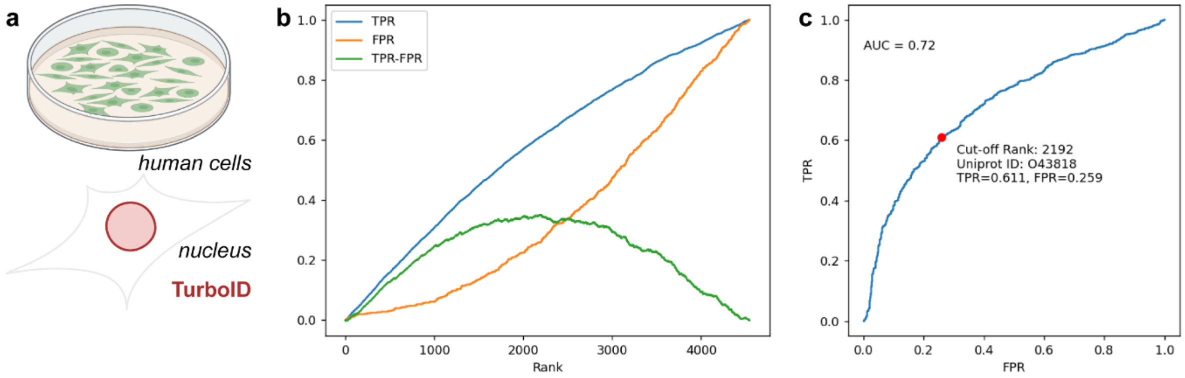

d Location: Panther GO Slim Cellular Component

Term	FDR
nucleus	3.848e-157
intracellular organelle	3.825e-101
organelle	1.882e-96
intracellular anatomical structure	4.842e-92
intracellular membrane-bounded organelle	1.846e-90
membrane-bounded organelle	1.183e-85
nuclear protein-containing complex	2.830e-84
nuclear lumen	6.668e-63
organelle lumen	1.155e-57
intracellular organelle lumen	1.271e-57

e Function: Panther GO Slim Biological Process

Term	FDR
nucleic acid metabolic process	7.936e-98
nucleobase-containing compound metabolic process	9.954e-88
cellular nitrogen compound metabolic process	9.833e-87
heterocycle metabolic process	5.512e-86
cellular aromatic compound metabolic process	1.942e-85
macromolecule metabolic process	1.068e-83
organic cyclic compound metabolic process	1.314e-83
RNA metabolic process	5.522e-77
gene expression	1.692e-73
nitrogen compound metabolic process	5.120e-70

f Pathway: Reactome

Term	FDR
Metabolism of RNA	1.534e-66
Gene expression (Transcription)	6.176e-45
Processing of Capped Intron-Containing Pre-mRNA	4.920e-40
mRNA Splicing	2.672e-35
Chromatin organization	3.015e-35
Chromatin modifying enzymes	3.768e-35
RNA Polymerase II Transcription	1.334e-34
mRNA Splicing - Major Pathway	1.948e-34
Cell Cycle	3.265e-26
DNA Repair	5.763e-23

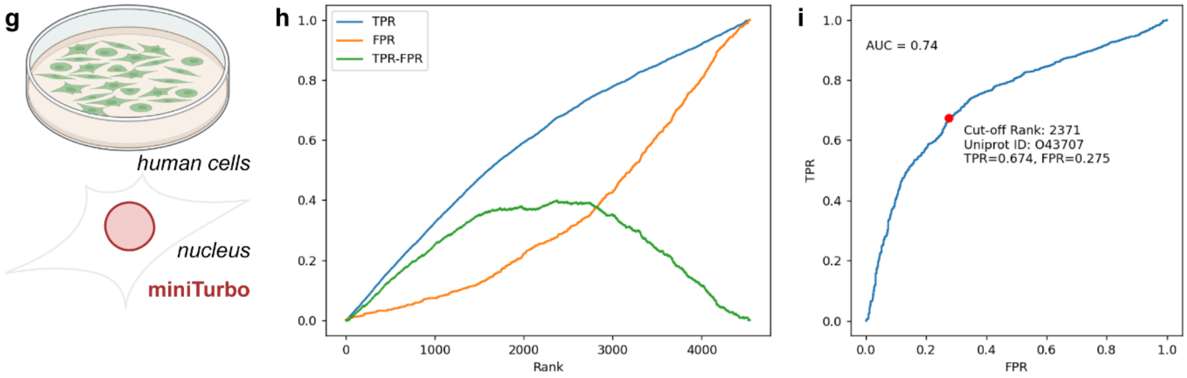

j Location: Panther GO Slim Cellular Component

Term	FDR
nucleus	8.559e-144
intracellular organelle	1.783e-98
intracellular membrane-bounded organelle	1.952e-95
organelle	3.908e-93
intracellular anatomical structure	2.237e-92
membrane-bounded organelle	4.483e-90
nuclear protein-containing complex	7.807e-80
nuclear lumen	1.449e-63
organelle lumen	3.772e-58
intracellular organelle lumen	4.149e-58

k Function: Panther GO Slim Biological Process

Term	FDR
nucleic acid metabolic process	1.864e-81
cellular nitrogen compound metabolic process	8.242e-76
nucleobase-containing compound metabolic process	2.061e-74
heterocycle metabolic process	2.204e-72
cellular aromatic compound metabolic process	3.869e-72
organic cyclic compound metabolic process	3.271e-71
macromolecule metabolic process	5.281e-67
gene expression	1.996e-64
RNA metabolic process	2.566e-64
nitrogen compound metabolic process	5.271e-58

l Pathway: Reactome

Term	FDR
Metabolism of RNA	2.280e-66
Gene expression (Transcription)	1.687e-34
Processing of Capped Intron-Containing Pre-mRNA	3.843e-34
rRNA processing	3.154e-30
rRNA processing in the nucleus and cytosol	3.807e-30
Chromatin organization	6.901e-29
Chromatin modifying enzymes	8.051e-29
mRNA Splicing	4.832e-28
Cell Cycle	7.489e-28
mRNA Splicing - Major Pathway	5.100e-27

Supplementary Figure 6. PEELing analysis of human nuclear proteomes.

(**a–f**) Analysis of a nuclear proteome of human embryonic kidney 293 cells, profiled by (Branon *et al.*, 2018) using the biotin ligase TurboID (**Supplementary File 5**). (**g–l**) Analysis of a nuclear proteome of human embryonic kidney 293 cells, profiled by (Branon *et al.*, 2018) using the biotin ligase miniTurbo (**Supplementary File 6**). (**b**,**h**) True positive rate (TPR, blue), false positive rate (FPR, orange), and their difference (TPR–FPR, green) plotted against ratio-based ranking (x-axis). Ratio used: **b**, 129:126; **h**, 130:131. (**c**,**i**) Receiver operating characteristic (ROC) curve. Red dot, cutoff position. AUC, area under the curve. Ratio used: **c**, 129:126; **i**, 130:131. (**d–f**, **j–l**) Protein ontology analyses of post-cutoff proteomes for localization (**d**,**j**), function (**e**,**k**), and pathway (**f**,**l**). FDR, false discovery rate.

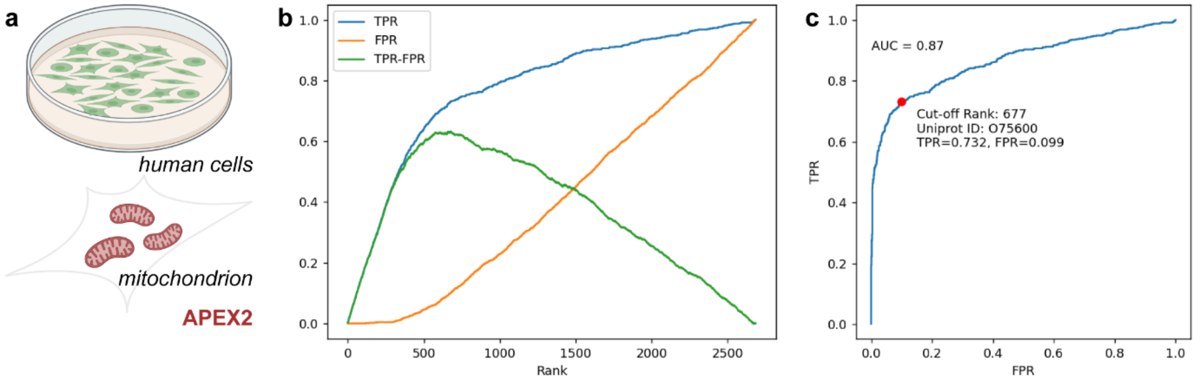

d Location: Panther GO Slim Cellular Component

Term	FDR
mitochondrion	3.962e-114
mitochondrial protein-containing complex	6.747e-31
mitochondrial matrix	9.464e-31
cytoplasm	1.678e-29
mitochondrial ribosome	2.793e-19
organellar ribosome	3.192e-19
mitochondrial inner membrane	5.763e-17
organelle inner membrane	6.703e-16
mitochondrial envelope	4.599e-15
ribosome	1.132e-14

e Function: Panther GO Slim Biological Process

Term	FDR
small molecule metabolic process	2.617e-48
carboxylic acid metabolic process	1.978e-35
oxoacid metabolic process	2.556e-35
organic acid metabolic process	2.130e-34
tRNA metabolic process	3.723e-27
cellular amino acid metabolic process	3.131e-25
ncRNA metabolic process	1.138e-23
organonitrogen compound biosynthetic process	1.133e-20
cellular amide metabolic process	3.687e-18
peptide metabolic process	6.093e-17

f Pathway: Reactome

Term	FDR
Mitochondrial translation	6.667e-59
Mitochondrial translation elongation	1.142e-56
Mitochondrial translation termination	1.713e-56
Mitochondrial translation initiation	1.815e-55
Translation	6.547e-55
Metabolism	4.840e-53
The citric acid (TCA) cycle and respiratory electron transport	1.738e-52
Respiratory electron transport, ATP synthesis by chemiosmotic coupling, and heat production by uncoupling proteins.	3.205e-30
Respiratory electron transport	2.834e-24
Pyruvate metabolism and Citric Acid (TCA) cycle	8.616e-23

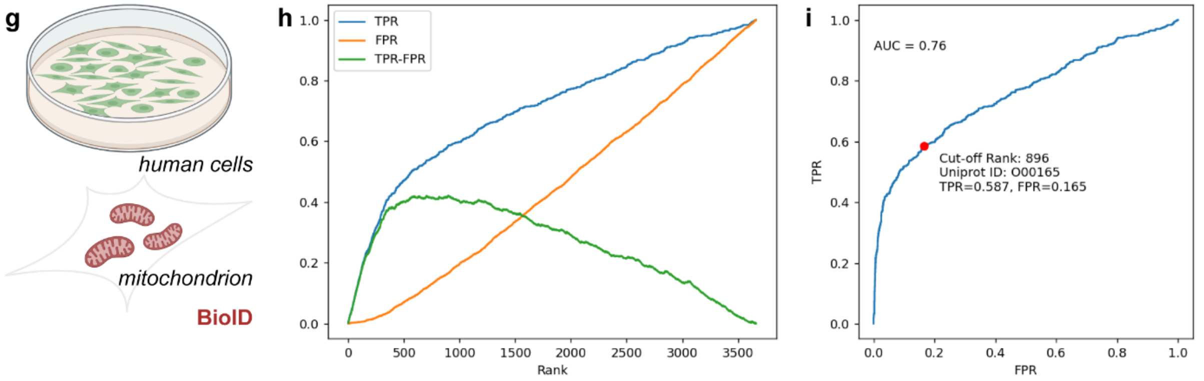

j Location: Panther GO Slim Cellular Component

Term	FDR
mitochondrion	1.634e-52
mitochondrial matrix	4.968e-23
mitochondrial protein-containing complex	2.993e-19
mitochondrial ribosome	1.735e-16
organellar ribosome	2.169e-16
cytoplasm	3.561e-12
ribosome	3.840e-12
intracellular anatomical structure	1.389e-10
ribosomal subunit	4.493e-10
mitochondrial large ribosomal subunit	4.962e-10

k Function: Panther GO Slim Biological Process

Term	FDR
small molecule metabolic process	2.949e-14
mitochondrial gene expression	1.708e-11
organonitrogen compound biosynthetic process	2.628e-11
cellular amide metabolic process	2.697e-11
translation	2.208e-10
peptide biosynthetic process	2.456e-10
translational elongation	2.650e-10
amide biosynthetic process	8.621e-10
carboxylic acid metabolic process	3.123e-9
peptide metabolic process	3.391e-9

l Pathway: Reactome

Term	FDR
Mitochondrial translation initiation	5.571e-51
Mitochondrial translation elongation	8.357e-51
Mitochondrial translation	1.016e-50
Mitochondrial translation termination	9.547e-50
Translation	1.862e-41
The citric acid (TCA) cycle and respiratory electron transport	6.021e-30
Respiratory electron transport	2.000e-18
Respiratory electron transport, ATP synthesis by chemiosmotic coupling, and heat production by uncoupling proteins.	2.205e-18
Complex I biogenesis	3.401e-14
Metabolism	1.587e-13

Supplementary Figure 7. PEELing analysis of human mitochondrial proteomes.

(**a–f**) Analysis of a mitochondrial nucleoid proteome of human embryonic kidney 293 cells, profiled by (Han *et al.*, 2017) using the peroxidase APEX2 (**Supplementary File 7**). (**g–l**) Analysis of a mitochondrial matrix proteome of human embryonic kidney 293 cells, profiled by (Branon *et al.*, 2018) using the biotin ligase BioID (**Supplementary File 8**). (**b**,**h**) True positive rate (TPR, blue), false positive rate (FPR, orange), and their difference (TPR–FPR, green) plotted against ratio-based ranking (x-axis). Ratio used: **b**, 126:128; **h**, 126:127. (**c**,**i**) Receiver operating characteristic (ROC) curve. Red dot, cutoff position. AUC, area under the curve. Ratio used: **c**, 126:128; **i**, 126:127. (**d–f**, **j–l**) Protein ontology analyses of post-cutoff proteomes for localization (**d**,**j**), function (**e**,**k**), and pathway (**f**,**l**). FDR, false discovery rate.

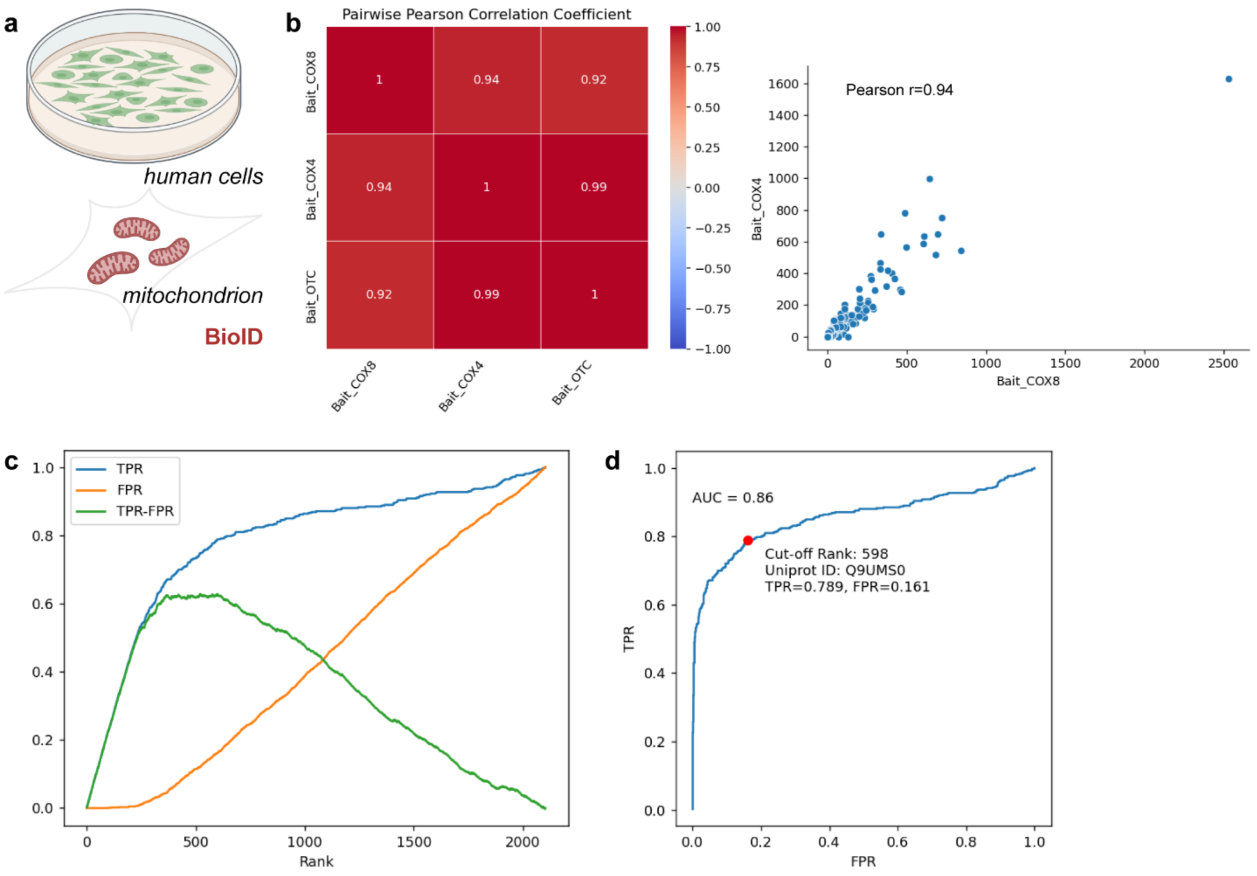

e Location: Panther GO Slim Cellular Component

Term	FDR
mitochondrion	1.736e-104
mitochondrial protein-containing complex	2.425e-47
mitochondrial matrix	4.550e-40
mitochondrial ribosome	4.503e-33
organellar ribosome	5.629e-33
ribosome	2.803e-26
cytoplasm	3.443e-21
mitochondrial inner membrane	5.613e-21
ribosomal subunit	6.314e-21
organelle inner membrane	2.011e-20

f Function: Panther GO Slim Biological Process

Term	FDR
cellular metabolic process	1.091e-34
translation	2.811e-30
amide metabolic process	3.434e-30
peptide biosynthetic process	4.012e-30
amide biosynthetic process	4.098e-30
peptide metabolic process	4.430e-28
cellular nitrogen compound biosynthetic process	4.795e-28
cellular biosynthetic process	2.003e-25
cellular nitrogen compound metabolic process	1.880e-24
organonitrogen compound biosynthetic process	2.223e-24

g Pathway: Reactome

Term	FDR
Mitochondrial translation	2.216e-93
Mitochondrial translation elongation	2.932e-92
Mitochondrial translation initiation	4.858e-91
Mitochondrial translation termination	6.477e-91
Translation	3.702e-82
The citric acid (TCA) cycle and respiratory electron transport	8.256e-63
Respiratory electron transport, ATP synthesis by chemiosmotic coupling, and heat production by uncoupling proteins.	1.858e-49
Respiratory electron transport	1.597e-41
Metabolism	1.162e-35
Complex I biogenesis	1.408e-29

Supplementary Figure 8. PEELing analysis of a label-free quantification mass spectrometry dataset.

(**a**) Analysis of a mitochondrial matrix proteome of human embryonic kidney 293 cells, profiled by (Antonicka *et al.*, 2020) using the biotin ligase BioID fused to three different bait proteins COX4, COX8, and OTC and label-free quantification mass spectrometry (**Supplementary File 9**). (**b**) Correlation plots and coefficients. (**c**) True positive rate (TPR, blue), false positive rate (FPR, orange), and their difference (TPR–FPR, green) plotted against the labelled-to-control ratio-based

ranking of the COX4 bait group (x-axis). (**d**) Receiver operating characteristic (ROC) curve, based on the labelled-to-control ratio-based ranking of the COX4 bait group. Red dot, cutoff position. AUC, area under the curve. (**e–g**) Protein ontology analyses for localization (**e**), function (**f**), and pathway (**g**). FDR, false discovery rate.

Supplementary Files

Supplementary File 1. A cell-surface proteome of mouse developing Purkinje cells (Shuster *et al.*, 2022). The authors used horseradish peroxidase (HRP) based cell-surface biotinylation and tandem mass tag (TMT) (Thompson *et al.*, 2003) based quantitative mass spectrometry. TMT tags 129C and 128C were used for labelled samples while 127C and 127N were used for non-labelled controls, producing four labelled-to-control ratios (129C:127C, 129C:127N, 128C:127C, and 128C:127N). Ratios are normalized and log_2-transformed.

Supplementary File 2. Two sets of random values, Random_1 (6th column) and Random_2 (7th column), were generated and added to the data of mouse Purkinje cell cell-surface proteome (Shuster *et al.*, 2022) (1st to 5th columns; from **Supplementary File 1**).

Supplementary File 3. Protein abundance data of the cell-surface proteome of mouse developing Purkinje cells (Shuster *et al.*, 2022), derived from the same mass spectrometry data as **Supplementary File 1**. The authors used horseradish peroxidase (HRP) based cell-surface biotinylation and tandem mass tag (TMT) (Thompson *et al.*, 2003) based quantitative mass spectrometry. TMT tags 129C and 128C were used for labelled samples. Abundance values are normalized and log_2-transformed.

Supplementary File 4. A cell-surface proteome of *Drosophila* mature olfactory projection neurons (Li *et al.*, 2020). The authors used horseradish peroxidase (HRP) based cell-surface biotinylation and tandem mass tag (TMT) (Thompson *et al.*, 2003) based quantitative mass spectrometry. TMT tags 126 and 129C were used for labelled samples while 127N and 128C were used for non-labelled controls, producing four labelled-to-control ratios (126:127N, 129C:127N, 126:128C, and 129C:128C). Ratios are normalized and log_2-transformed.

Supplementary File 5. A nuclear proteome of human embryonic kidney 293 cells (Branon *et al.*, 2018). The authors used the biotin ligase TurboID for spatially-resolved biotinylation in the nucleus and tandem mass tag (TMT) (Thompson *et al.*, 2003) for quantitative mass spectrometry. Two labelled-to-control ratios were provided: TMT tag 128:129 from Replicate 1 and 129:126 from Replicate 2. Ratios are normalized and log_2-transformed.

Supplementary File 6. A nuclear proteome of human embryonic kidney 293 cells (Branon *et al.*, 2018). The authors used the biotin ligase miniTurbo for spatially-resolved biotinylation in the nucleus and tandem mass tag (TMT) (Thompson *et al.*, 2003) for quantitative mass spectrometry.

Two labelled-to-control ratios were provided: TMT tag 130:131 from Replicate 1 and 127:126 from Replicate 2. Ratios are normalized and $\log_2$-transformed.

Supplementary File 7. A mitochondrial nucleoid proteome of human embryonic kidney 293 cells (Han *et al.*, 2017). The authors used the peroxidase APEX2 for spatially-resolved biotinylation in the mitochondrion and tandem mass tag (TMT) (Thompson *et al.*, 2003) for quantitative mass spectrometry. Two labelled-to-control ratios were provided: TMT tag 126:128 from Replicate 1 and 129:131 from Replicate 2. Ratios are normalized and $\log_2$-transformed.

Supplementary File 8. A mitochondrial matrix proteome of human embryonic kidney 293 cells (Branon *et al.*, 2018). The authors used the biotin ligase BioID for spatially-resolved biotinylation in the mitochondrion and tandem mass tag (TMT) (Thompson *et al.*, 2003) for quantitative mass spectrometry. Two labelled-to-control ratios were provided: TMT tag 126:127 from Replicate 1 and 130:131 from Replicate 2. Ratios are normalized and $\log_2$-transformed.

Supplementary File 9. A mitochondrial matrix proteome of human embryonic kidney 293 cells (Antonicka *et al.*, 2020). The authors used the biotin ligase BioID fused to three different bait proteins COX4, COX8, and OTC for spatially-resolved biotinylation in the mitochondrion and label-free quantification mass spectrometry. One labelled-to-control ratio was provided for each bait.

Supplementary File 10. True positive (TP) reference for the cell surface, as of July 2, 2023.

Supplementary File 11. 10%-coverage TP reference for the cell surface, produced by randomly removing 90% of genes from **Supplementary File 10**.

Supplementary File 12. False positive (FP) reference for the cell surface, as of July 2, 2023.

Supplementary File 13. 10%-coverage FP reference for the cell surface, produced by randomly removing 90% of genes from **Supplementary File 12**.

Supplementary File 14. True positive (TP) reference for the nucleus, as of July 2, 2023.

Supplementary File 15. False positive (FP) reference for the nucleus, as of July 2, 2023.

Supplementary File 16. True positive (TP) reference for the mitochondrion, as of July 2, 2023.

Supplementary File 17. False positive (FP) reference for the mitochondrion, as of July 2, 2023.
